# Inhibition of Calcium Signaling Prevents Exhaustion and Enhances Anti‐Leukemia Efficacy of CAR‐T Cells via SOCE‐Calcineurin‐NFAT and Glycolysis Pathways

**DOI:** 10.1002/advs.202103508

**Published:** 2022-01-14

**Authors:** Mi Shao, Xinyi Teng, Xin Guo, Hao Zhang, Yue Huang, Jiazhen Cui, Xiaohui Si, Lijuan Ding, Xiujian Wang, Xia Li, Jimin Shi, Mingming Zhang, Delin Kong, Tianning Gu, Yongxian Hu, Pengxu Qian, He Huang

**Affiliations:** ^1^ Bone Marrow Transplantation Center The First Affiliated Hospital Zhejiang University School of Medicine Hangzhou Zhejiang 310003 China; ^2^ Institute of Hematology Zhejiang University Hangzhou Zhejiang 310030 China; ^3^ Zhejiang Province Engineering Laboratory for Stem Cell and Immunity Therapy Hangzhou Zhejiang 310030 China; ^4^ Liangzhu Laboratory Zhejiang University Medical Center Hangzhou Zhejiang 311121 China; ^5^ Department of Hematology The Third Affiliated Hospital of Wenzhou Medical University Wenzhou Zhejiang 315200 China; ^6^ Center of Stem Cell and Regenerative Medicine Zhejiang University School of Medicine Hangzhou Zhejiang 310030 China

**Keywords:** calcium signaling, chimeric antigen receptor T, exhaustion, glycolysis, store‐operated calcium entry

## Abstract

Chimeric antigen receptor (CAR) T cells are potent agents for recognizing and eliminating tumors, and have achieved remarkable success in the treatment of patients with refractory leukemia and lymphoma. However, dysfunction of T cells, including exhaustion, is an inevitable obstacle for persistent curative effects. Here, the authors initially found that calcium signaling is hyperactivated via sustained tonic signaling in CAR‐T cells. Next, it is revealed that the store‐operated calcium entry (SOCE) inhibitor BTP‐2, but not the calcium chelator BAPTA‐AM, markedly diminishes CAR‐T cell exhaustion and terminal differentiation of CAR‐T cells in both tonic signaling and tumor antigen exposure models. Furthermore, BTP‐2 pretreated CAR‐T cells show improved antitumor potency and prolonged survival in vivo. Mechanistically, transcriptome and metabolite analyses reveal that treatment with BTP‐2 significantly downregulate SOCE‐calcineurin‐nuclear factor of activated T‐cells (NFAT) and glycolysis pathways. Together, the results indicate that modulating the SOCE‐calcineurin‐NFAT pathway in CAR‐T cells renders them resistant to exhaustion, thereby yielding CAR products with enhanced antitumor potency.

## Graphical abstract

This work reports that calcium signaling is hyperactivated via sustained tonic signaling in chimeric antigen receptor (CAR) T cells. The store‐operated calcium entry (SOCE) inhibitor BTP‐2 markedly diminishes exhaustion and terminal differentiation both in tonic signaling and tumor antigen exposure models and enhances antitumor potency in vivo. Mechanistically, BTP‐2 treatment significantly downregulates SOCE‐calcineurin‐nuclear factor of activated T‐cells and glycolysis pathways.

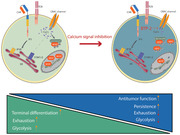

## 1 Introduction

The development of numerous immunotherapy strategies for cancer treatment has accelerated in recent years. Among these therapies, chimeric antigen receptor (CAR)‐modified T cell therapies have received considerable attention. In particular, CD19‐specific CAR‐T therapy has been demonstrated to achieve a marked therapeutic response in patients with refractory or relapsed leukemia^[^ ^1^ ^]^ and lymphoma^[^ ^2^ ^]^ and was recently approved by the US Food and Drug Administration.^[^ ^3^ ^]^ However, CAR‐T therapy has exhibited high tumor recurrence rates, ranging from 21% to 49%,^[^ ^1^, ^2^ ^]^ and has been less successful in treating solid tumors owing to multiple obstacles, including tumor escape consequent to the loss or mutation of the targeted antigen, the immunosuppressive tumor microenvironment, and intrinsic T cell dysfunction such as T cell exhaustion.^[^ ^4^ ^]^ T cell exhaustion is characterized by high expression of inhibitory receptors as well as transcriptional and epigenetic alterations.^[^ ^5^ ^]^ Additionally, T cell exhaustion has been increasingly reported as a cause of CAR‐T cell dysfunction.^[^ ^4^, ^5^ ^]^ Excessive T cell activation by sustained tonic signaling or tumor antigen stimulation drives exhaustion and differentiation of T cells,^[^ ^5^, ^6^ ^]^ including CAR‐T cells.^[^ ^7^ ^]^ Alternatively, as AP‐1/bZIP family and NR4A transcription factors constitute major factors driving exhaustion‐associated gene expression, c‐Jun overexpression or NR4A‐deficiency can render CAR‐T cells exhaustion‐resistant.^[^ ^8^ ^]^ Moreover, methods of restricting or transient inhibiting CAR signaling, such as mutating immunoreceptor tyrosine‐based activation motif domains, inducing CAR protein degradation, or using dasatinib could redirect CAR T cell fate away from exhaustion toward a memory‐like state.^[^ ^9^ ^]^

Ionized calcium (Ca^2+^) operates as a universal intracellular second messenger in virtually all eukaryotic cells, including immune cells. Ca^2+^ signals are crucial for the proper activation of lymphocytes, their differentiation and effector functions.^[^ ^10^ ^]^ The main source of Ca^2+^ signals in T cells is store‐operated calcium entry (SOCE), which is mediated by the activation of Ca^2+^ release‐activated Ca^2+^ (CRAC) channels.^[^ ^11^ ^]^ The intensity and duration of the Ca^2+^ signal generated via this mechanism affect T cell effector function and fate determination upon activation, in collaboration with other pathways, such as the T cell receptor pathway.^[^ ^12^ ^]^ Thus, we hypothesized that modulation of cytosolic calcium in CAR‐T cells would prevent exhaustion while reinvigorating T cell function. In this study, we showed that inhibition of SOCE rendered CAR‐T cells resistant to the development of intrinsic dysfunction, as demonstrated by decreased phenotypic hallmarks of exhaustion, diminished terminal differentiation, increased functional capacity, and improved antitumor efficacy in both in vitro and in vivo models, and that this resistance occurred through the SOCE‐calcineurin‐nuclear factor of activated T‐cells (NFAT) pathway.

## 2 Results

### 2.1 Calcium Signaling is Hyperactivated via Sustained Tonic Signaling in CAR‐T Cells

**Figure 1:**
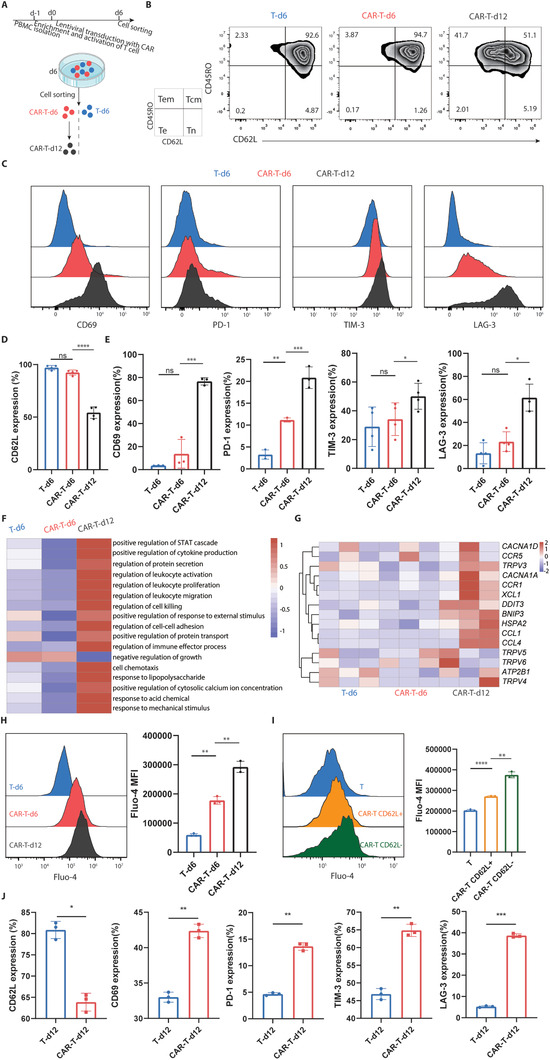
Calcium signal was enhanced via tonic signaling. A) Experimental design: T cells were isolated from PBMCs, enriched, and activated at day ‐1. 4‐1BB CAR lentivirus was transduced at day 0. CAR‐T cells were cultured in vitro and sorted at day 5. Unmodified T cells, CAR‐T cells at day 6, and CAR‐T cells at day 12 were collected for FACS and bulk RNA sequencing. Flow cytometric analysis of the expression of B,D) effector or memory markers (CD62L and CD45RO) and C,E) activation marker (CD69) and inhibitory markers (PD‐1, TIM‐3, LAG‐3) on T cells. F) GO‐term enrichment analysis showing the list of top signaling pathways in each group. G) The heatmap and clustering of fragments per kilobase per million (FPKM) of the calcium‐related genes in each group. Flow cytometric analysis of the concentration of intracellular calcium in H) T cells at different culture times and I) CAR‐T cells of different phenotypes. J) Flow cytometric analysis of the expression of differentiation (CD62L), activation (CD69), and inhibitory (PD‐1, TIM‐3, and LAG‐3) markers on T cells. Data are reported as the means ± SEMs. *n =* 3 or more independent biological replicates, presented as individual points. *p < 0.05, **p < 0.01, and ***p ≤ 0.001 (one‐way ANOVA with Dunnett post‐hoc test; comparing *n =* 3 or more in vitro biological replicates per group).

Early exhaustion in CAR‐T cells, especially those expressing the CD28 costimulatory domain, was recently reported to result from sustained tonic signaling triggered by antigen‐independent clustering of CAR scFvs.^[^ ^4a^ ^]^ Compared to CD19‐CD28 CAR, CD19‐targeted CAR incorporating with the 4‐1BB costimulation domain showed a lower tendency toward exhaustion during ex vivo expansion, although downstream differentiation was still observed with a prolonged culture time.^[^ ^13^ ^]^ Here, we employed 4‐1BB CD19‐targeted CAR‐T cells as a model to study CAR‐T cell exhaustion because CAR‐T cells could be readily collected at different stages of exhaustion (**Figure** **1**A). In contrast to unmodified T cells (cultured for 6 days in vitro) and short‐term cultured (6 days) CAR‐T cells, long‐term cultured (12 days) CAR‐T cells exhibited profound features of exhaustion, including increased expression of inhibitory receptors (PD‐1, TIM‐3, and LAG‐3) and exaggerated terminal differentiation (marked by the expression of CD62L and CD45RO), and showed increased activation (indicated by CD69 expression) (Figure 1B–E).

To elucidate the underlying mechanisms responsible for CAR‐T cell exhaustion, we then sorted CAR‐T cells at different time points and performed RNA‐Seq. Gene ontology (GO) enrichment analysis revealed that the differentially expressed genes (DEGs) of long‐term cultured CAR‐T cells were predominantly enriched in pathways involved in cell proliferation, activation, killing, migration, adhesion, and cytokine production, which are essential characteristics of CAR‐T cells. Moreover, the pathway for regulation of the cytosolic calcium ion concentration was significantly upregulated in the CAR‐T‐d12 group (Figure 1F). In particular, we found that the expression of calcium signaling genes (e.g., *CACNA1A*, *CACNA1D*, *TRPV3*, *TRPV4*, *TRPV5*, *TRPV6*, *DDIT3*, and *ATP2B1*) were markedly increased in the CAR‐T‐d12 group (Figure 1G). Next, we measured the cytosolic calcium concentration in CAR‐T cells at different culture times (6 and 12 days) or at different stages (memory or effector). Cytosolic calcium concentrations were significantly increased in CAR‐T cells cultured for 12 days or at the effector (CD62L negative) stage compared to that in the control (Figure 1H,I). To exclude the influence of the initial anti‐CD3/CD28 stimulation or extended culture in extra IL‐2, we further compared unmodified T cells (cultured for 12 days in vitro) and long‐term cultured (12 days) CAR‐T cells. CAR‐T cells exhibited higher levels of activation and exhaustion markers and a decreased proportion of cells bearing naïve and central memory phenotypes compared to those of unmodified T cells (Figure 1J), which confirms that the above differences are mainly caused by tonic signaling.

### 2.2 The SOCE Inhibitor BTP‐2 Inhibits Calcium Signaling and Reduced Excessive Activation of CAR‐T Cells

**Figure 2:**
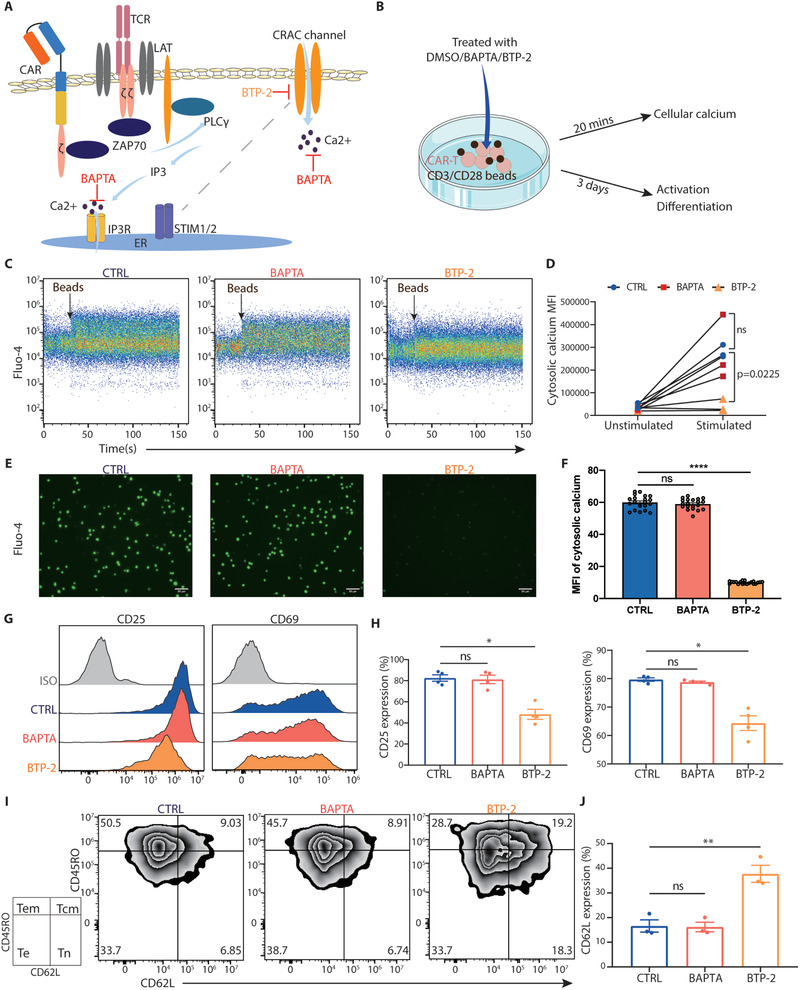
The SOCE inhibitor BTP‐2 reduced excessive activation of CAR‐T cells. A) Schematic diagram of the mechanism of different calcium inhibitors. B) Experimental design: CAR‐T cells were stimulated using anti‐CD3/CD28 beads and treated with DMSO/BAPTA/BTP‐2 on days 6–9 following activation or FACS. C–F) Flow cytometric analysis and fluorescence imaging of the intracellular calcium concentration of each group. Histograms and plots of the representative donor are shown. Flow cytometric analysis of the expression of G,H) activation (CD25 and CD69) and I,J) memory (CD62L and CD45RO) markers on CAR+ T cells. Data are reported as the means ± SEMs. *n =* 3 or more independent biological replicates, presented as individual points. *p < 0.05, **p < 0.01, and ***p ≤ 0.001 (one‐way ANOVA with Dunnett post‐hoc test; comparing *n =* 3 or more in vitro biological replicates per group).

Because we observed that the hyperactivated calcium signaling correlated with the activation, differentiation, and exhaustion of CAR‐T cells, we hypothesized that blocking calcium signaling might prevent CAR‐T cells from excessive activation. To test this hypothesis, we initially used anti‐CD3/CD28 beads to induce T‐cell activation,^[^ ^14^ ^]^ and simultaneously treated CAR‐T cells with two types of calcium inhibitors, BAPTA‐AM (an intracellular Ca^2+^ chelator) and BTP‐2 (a SOCE inhibitor), to measure the intracellular Ca^2+^ concentration and cell phenotype at different time points (**Figure** **2**A,B). We found that BAPTA‐AM was ineffective at preventing intracellular accumulation of Ca^2+^, which may be due to the entry of extracellular calcium (e.g., via SOCE) exceeding the buffering capacity of BAPTA‐AM.^[^ ^15^ ^]^ In stark contrast, BTP‐2 strongly inhibited the influx of calcium ions following anti‐CD3/CD28 beads stimulation (Figure 2C–F). To further assess whether regulating the intracellular Ca^2+^ concentration affects the terminal differentiation caused by T cell hyperactivation, we sorted 4‐1BB CAR‐T cells by flow cytometry and simultaneously treated them with anti‐CD3/CD28 beads and the two calcium inhibitors for 48 h. Consistent with our hypothesis, we found that 4‐1BB CAR‐T cells treated with BTP‐2, but not BAPTA‐AM, were maintained in the states of naïve T cells (Tn) and central memory T cells (Tcm) T cells and exhibited relatively low levels of activation (Figure 2G–J). Moreover, we also treated CD28 CAR‐T cells with the two calcium inhibitors for 48 h and obtained similar results (Figure S1, Supporting Information).

### 2.3 BTP‐2 Prevents CAR‐T Cells from Exhaustion during the Prolonged In Vitro Culture

**Figure 3:**
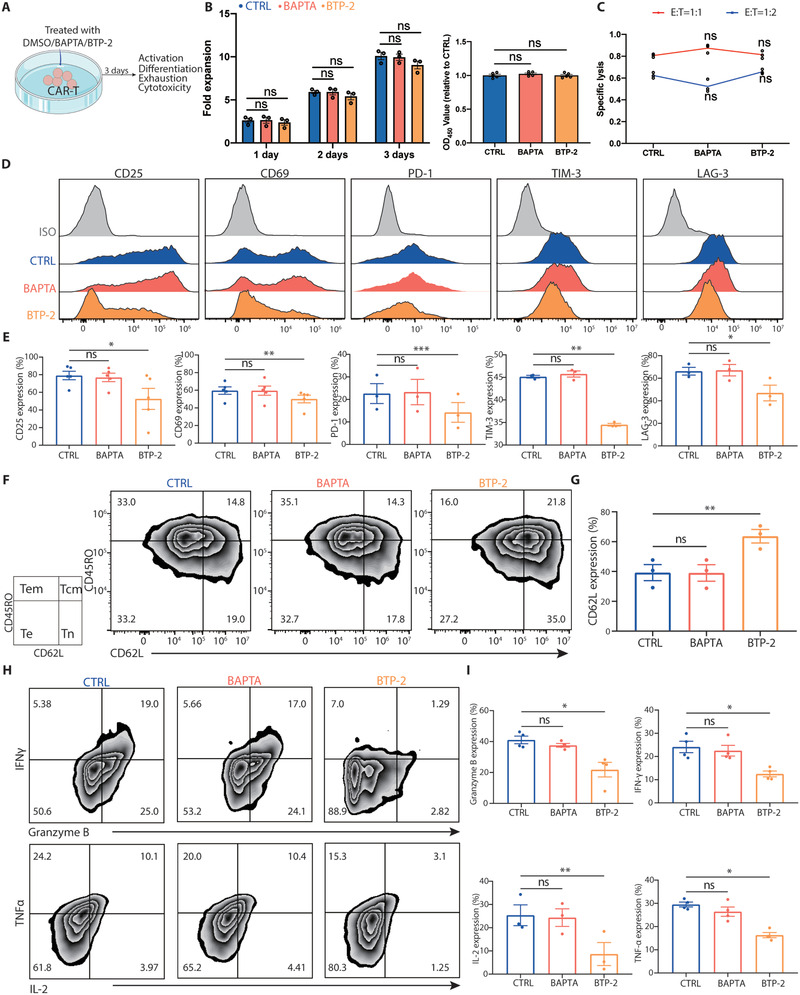
BTP‐2 prevents CAR‐T cell exhaustion in vitro. A) Experimental design: CAR‐T cells were cultured in vitro and treated with DMSO/BAPTA/BTP‐2 at day 9–12 for three consecutive days. B) The number of CAR‐T cells was calculated using cell counts on days 1, 2, and 3, respectively, and CCK8 was calculated on day 3. C) Lysis of target cells measured at 4 h. Effector‐to‐target (E:T) ratios were 1:1 (red line) or 1:2 (blue line). Flow cytometric analysis of the expression of D,E) activation (CD25 and CD69), inhibitory receptor (PD‐1, TIM‐3, and LAG‐3) and F,G) differentiation status (CD62L and CD45RO) markers. H,I) Flow cytometric analysis of the expression of intracellular cytokines (granzyme B, IFN‐*γ*, IL‐2, and TNF‐*α*). Data are reported as the means ± SEMs. *n =* 3 or more independent biological replicates, presented as individual points. *p < 0.05, **p < 0.01, and ***p ≤ 0.001 (one‐way ANOVA with Dunnett post‐hoc test; comparing *n =* 3 or more in vitro biological replicates per group).

We further reasoned that excessive intracellular calcium accumulation led to excessive activation of CAR‐T cells, which would induce differentiation and exhaustion during the prolonged in vitro culture. To test whether inhibiting calcium signaling might affect CAR‐T cell proliferation and killing ability, we treated 4‐1BB CAR‐T cells with different concentrations of BAPTA‐AM and BTP‐2 for three consecutive days and found that low concentrations of the two inhibitors did not affect the proliferation or specific tumor lysis capacity of the cells (**Figure** **3**A–C; Figure S2A,B, Supporting Information). Notably, CAR‐T cells treated with 1.5 µm BTP‐2 displayed markedly diminished expression of activation markers (CD25 and CD69) and exhaustion markers (PD‐1, TIM‐3, and LAG‐3), whereas they exhibited an increased proportion of cells bearing naïve and central memory phenotypes compared with those of control and BAPTA‐AM‐treated CAR‐T cells (Figure 3D–F). Consistent with this, the results of flow cytometry analysis revealed that the expression of granzyme B, IFN‐*γ*, IL‐2, and TNF‐*α* was significantly decreased in the BTP‐2‐treated group, indicating a low level of activation (Figure 3H,I). We next cocultured CAR‐T cells with Nalm6 cells in the presence of 1.5 µm BTP‐2 and found that although BTP‐2 at low concentration weakened the proliferation and killing ability, inhibited the activation and reduced the cytokine production of CAR‐T cells, the expression of exhaustion markers was profoundly reduced and CD62L expression was increased (Figure S2C–G, Supporting Information). Furthermore, the results obtained upon utilization of CD28 CAR‐T cells as an alternative model were consistent with those from 4‐1BB CAR‐T cells (Figure S3A–C, Supporting Information). Together, these data demonstrated that application of the SOCE inhibitor BTP‐2 at low concentration reduced exhaustion and terminal differentiation, while sparing the proliferation and killing ability of CAR‐T cells during the prolonged in vitro culture.

### 2.4 Pretreatment of CAR‐T Cells with BTP‐2 Enhances the In Vivo Antitumor Activity

**Figure 4:**
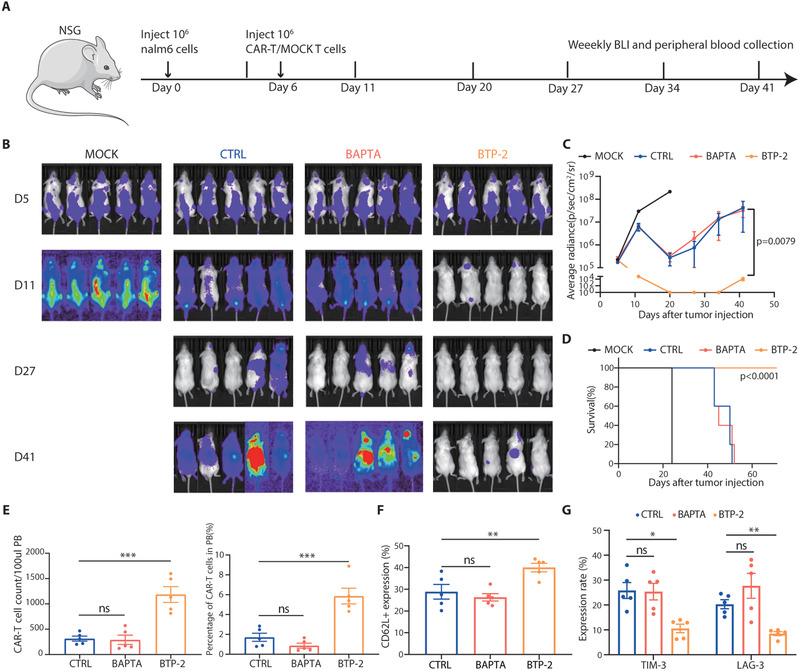
BTP‐2 enhances antitumor activity of CAR‐T cells in vivo. A) Treatment schedule and experimental set‐up. NSG mice received either 1 × l0^6^ Nalm6 cells on day 0 and either untransduced T cells (MOCK) or 4‐1BB CD19‐CAR‐T cells on day 6. B) D5‐41 bioluminescence imaging of tumor growth (*n =* 5 mice/group). C) The dorsal BLI signal is displayed for individual mice in each treatment group. D) Kaplan–Meyer survival plot for mice receiving mock T cells or CAR‐T cells pretreated with different calcium inhibitors (*n =* 5) (statistical analysis by Mantel–Cox test, *p* < 0.0001). CAR‐T cells were isolated from peripheral blood on day 8 and analyzed by flow cytometry for E) count and percentage, F) differentiation status (CD62L), and G) expression of inhibitory receptors (TIM‐3 and LAG‐3). Data are reported as the means ± SEMs. *n =* 5 independent biological replicates, presented as individual points. *p < 0.05, **p < 0.01, and ***p ≤ 0.001 (one‐way ANOVA with Dunnett post‐hoc test; comparing *n =* 5 independent replicates per group).

Using a luciferase/GFP/Nalm6‐bearing mouse leukemia model (**Figure** **4**A), we tested the functional superiority of BTP‐2‐pretreated CAR‐T cells, which yielded a significantly lower tumor burden and longer‐term remission in all treated mice and prolonged survival compared to those of the control group (Figure 4B–D). Moreover, flow cytometry of peripheral blood collected at 8 days following CAR‐T cell infusion revealed that CAR‐T cells pretreated with the SOCE inhibitor BTP‐2 significantly increased homeostatic expansion in vivo (Figure 4E). To investigate the functional basis underlying these major differences in antitumor efficacy, we examined the exhaustion markers and memory subtypes of CAR‐T cells in the different treatment groups. CAR‐T cells in the BTP‐2 group exhibited a higher percentage of CD62L‐positive cells (Tn and Tcm) and diminished expression of inhibitory receptors (Figure 4F,G), illustrating the benefit of reducing calcium signaling within CAR‐T cells expressing a 4‐1BB costimulatory domain.

### 2.5 BTP‐2 Reverses the CAR‐T Cell Differentiation and Exhaustion Induced by Tumor Antigen Exposure

**Figure 5:**
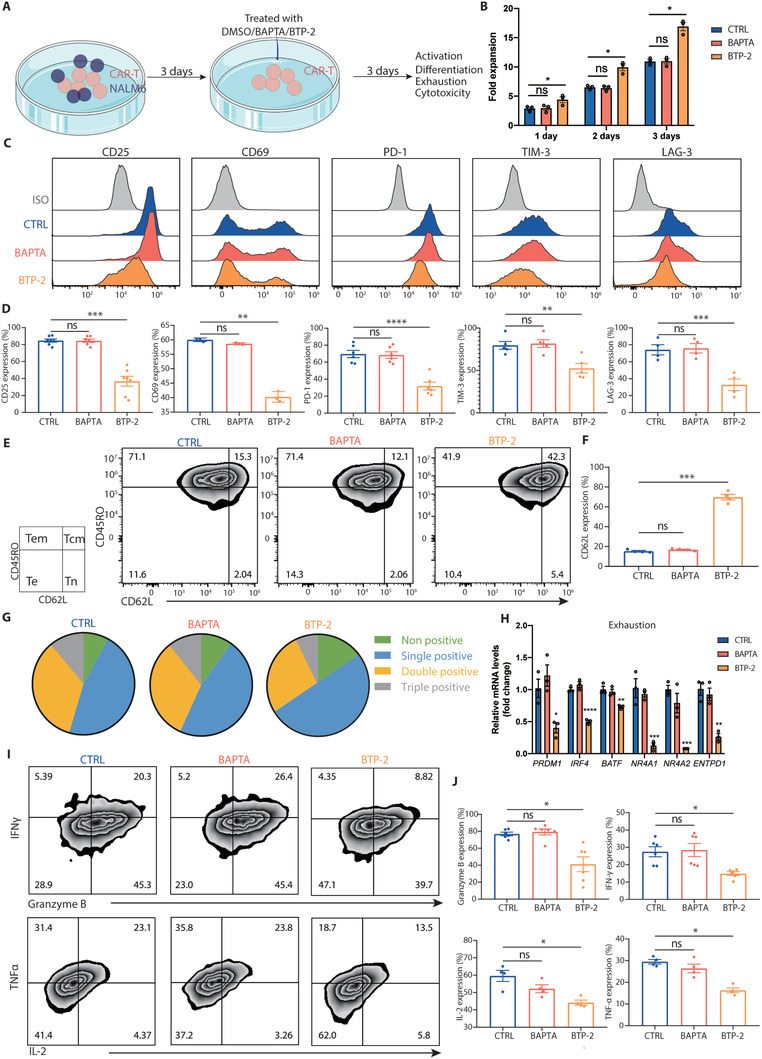
BTP‐2 reverses CAR‐T cell differentiation and exhaustion induced by tumor antigen exposure A) Experimental design: CAR‐T cells were cocultured with Nalm6 cells at a ratio of 1:1 for 72 h. The remaining CAR‐T cells were collected and treated with DMSO/BAPTA/BTP‐2 for 3 days. B) The number of CAR‐T cells was calculated using cell counts on days 1, 2, and 3. Flow cytometric analysis of the expression of C,D) activation (CD25 and CD69), inhibitory receptor (PD‐1, TIM‐3, and LAG‐3) and E,F) differentiation status (CD62L and CD45RO) markers. G) Patterns of expression and co‐expression of the inhibitory molecules PD‐1, TIM‐3, and LAG‐3. The proportions of non‐positive (PD1^‐^TIM3^‐^LAG3^‐^), single‐positive (PD1^+^TIM3^‐^LAG3^‐^ or PD1^‐^TIM3^+^LAG3^‐^ or PD1^‐^TIM3^‐^LAG3^+^), double‐positive (PD1^+^TIM3^+^LAG3^‐^ or PD1^+^TIM3^‐^LAG3^+^ or PD1^‐^TIM3^+^LAG3^+^), and triple‐positive (PD1^+^TIM3^+^LAG3^+^) are depicted, which were detected by FACS. H) mRNA level of exhaustion‐related transcription factors in different groups. I,J) Flow cytometric analysis of the expression of intracellular cytokines (granzyme B, IFN‐*γ*, IL‐2, and TNF‐*α*). Data are reported as the means ± SEMs. *n =* 3 or more independent biological replicates, presented as individual points. *p < 0.05, **p < 0.01, ***p ≤ 0.001, and ****p ≤ 0.0001 (one‐way ANOVA with Dunnett post‐hoc test; comparing *n =* 3 or more in vitro biological replicates per group).

After killing target tumor cells, the remaining CAR‐T cells enter into a hyporesponsive (exhausted or dysfunctional) state, which is characterized by the upregulation of inhibitory receptors and loss of effector function.^[^ ^8^, ^16^ ^]^ After proving that the SOCE inhibitor BTP‐2 can reduce the exhaustion and differentiation of CAR‐T cells caused by tonic signaling, we asked whether the BTP‐2 could prevent or reverse the exhaustion and terminal differentiation of CAR‐T cells caused by tumor antigens by constructing an in vitro killing model of CAR‐T cells cocultured with Nalm6 cells.^[^ ^17^ ^]^ We collected the remaining CAR‐T cells and treated them with calcium inhibitors for 72 h (**Figure** **5**A). Notably, BTP‐2 treatment significantly increased the number of CAR‐T cells and simultaneously reduced their activation (as indicated by assessment of activation markers and cytokine production) and exhaustion levels (as indicated by assessment of three inhibitory receptors), and increased the proportion of naïve and central memory CAR‐T cells (Figure 5B–F,I,J). Moreover, we analyzed the patterns of expression and co‐expression of the inhibitory molecules PD‐1, TIM‐3, and LAG‐3, and found that the populations of double‐positive and triple‐positive cells in the BTP‐2 group were significantly smaller than those in the control group, whereas the populations of non‐positive and single‐positive cells were significantly larger than those in the control group (Figure 5G). Additionally, we analyzed exhaustion‐related transcription factors and found that the BTP‐2 markedly decreased the mRNA levels of *PRDM1*, *IRF4*, *BATF*, *NR4A1*, *NR4A2*, and *ENTPD1* (Figure 5H). Furthermore, we examined the apoptosis of CAR‐T cells, and found that BTP‐2 treatment had no significant effect (Figure S4A,B, Supporting Information). The change in the proportion of CD4 and CD8 subsets was also analyzed, which indicated that the BTP‐2 increased the ratio of CD8 to CD4 in CAR‐T cells (Figure S4C,D, Supporting Information). Additionally, the effect of BTP‐2 on differentiation was significant in both CD4‐CAR‐T cells and CD8‐CAR‐T cells (Figure S4E,F, Supporting Information).

### 2.6 BTP‐2 Impedes CAR‐T Cell Exhaustion via the Calcium‐Calcineurin‐NFAT and Glycolysis Pathways

**Figure 6:**
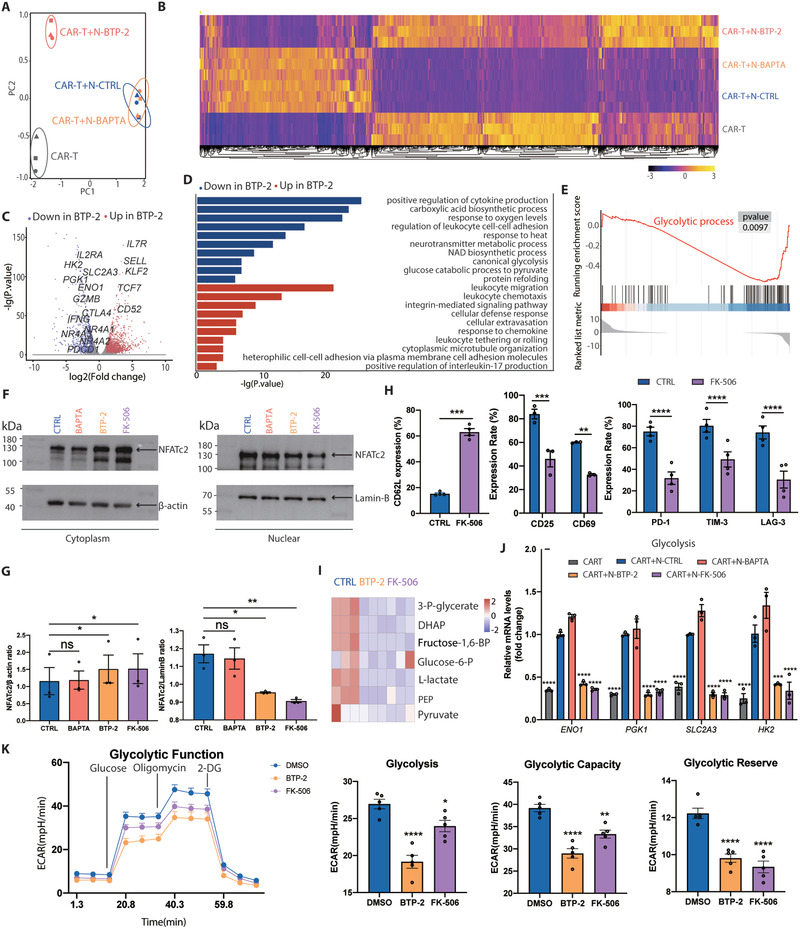
BTP‐2 impedes CAR‐T cell exhaustion through the calcium‐calcineurin‐NFAT and glycolysis pathways. A) Principal component analysis of CAR‐T cells in different groups. B) Heatmap showing the FPKM values of a total of 2061 DEGs in comparisons with the control group. C) Volcano plot depicting the significant DEGs in the BTP‐2 group compared with the control. The red or blue dots respectively represent the genes with up‐ or down‐regulated expression with fold change >2 or <0.5, respectively, and −log_10_ *p*‐value >2. D) The red or blue bars illustrate the pathways in which the up‐ or down‐regulated genes in the BTP‐2 group, respectively, are over‐represented. E) GSEA plot of which from GO gene sets. Enrichment plot of the glycolytic process from the GO gene set obtained performing GSEA. F,G) For western blot analysis of NFATc1, Nalm6 stimulated CAR‐T cells were treated with DMSO/BAPTA/BTP‐2/FK‐506 for 3 days and the cytoplasmic proteins and nuclear proteins were separated. Quantitative analysis of western blot data obtained in *n =* 3 experiments is shown, normalized to *β*‐Actin. H) Flow cytometric analysis of the expression of differentiation status (CD62L and CD45RO), activation (CD25 and CD69), and inhibitory receptor (PD‐1, TIM‐3, and LAG‐3) markers in FK‐506 treated CAR‐T cells and CTRL CAR‐T cells. I) Metabolomic analysis for metabolites in the EMP in CAR‐T cells treated with DMSO/BTP‐2/FK‐506. J) mRNA level of glycolysis‐related enzymes in different groups. Data are presented as the means ± SEM (3 or more independent experiments). K) The extracellular acidification rate (ECAR) was measured in real time in an XFe96 analyzer after injection of glucose, oligomycin, and 2‐deoxy‐D‐glucose (2DG). Graphical analysis of glycolysis, glycolytic capacity, and glycolytic reserve (*n =* 5). Data reported as means ± SEMs. *n =* 3 or more independent biological replicates, presented as individual points. *p < 0.05, **p < 0.01, ***p ≤ 0.001, and ****p ≤ 0.0001 (G, J, and K: one‐way ANOVA with Dunnett post‐hoc test, H: two‐tailed paired Student's *t*‐tests, comparing *n =* 3 or more in vitro biological replicates per group).

To further investigate the underlying mechanisms of BTP‐2 in CAR‐T cells, we performed RNA‐Seq in CAR‐T cells that were cocultured with Nalm6 and then treated with DMSO, BAPTA‐AM, or BTP‐2. Unbiased principal component analysis (PCA) showed that BTP‐2‐treated CAR‐T cells were closer to unstimulated CAR‐T cells, which were distant from the BAPTA‐AM‐treated group and DMSO‐treated groups (**Figure** **6**A). Consistent with this, the transcriptome profile of the BTP‐2‐treated CAR‐T cells was similar to that of the unstimulated CAR‐T cells (Figure 6B). In particular, analysis of the DEGs between the BTP‐2‐treated group and the DMSO‐treated control group and revealed that the expression of memory‐associated transcription factors (*TCF7, KLF2*, and *IL7R*) and naïve/memory‐associated cell surface markers (*CCR7* and *SELL*) was markedly upregulated in BTP‐2‐treated CAR‐T cells. In contrast, the expression of exhaustion‐related regulators (*NR4A1*, *NR4A2*, and *NR4A3*) and inhibitory receptors (*PDCD1* and *CTLA4*) was significantly downregulated in BTP‐2‐treated (Figure 6C). Interestingly, we found that expression of the glucose transporter GLUT3 (*SLC2A3*) and glycolytic enzymes (*ENO1*, *HK2*, and *PGK1*) was reduced in BTP‐2‐treated CAR‐T cells. To determine the biological functions influenced by BTP‐2, we performed GO enrichment analysis. Genes downregulated in the BTP‐2 group were predominantly enriched in the regulation of cytokine production, protein refolding, and regulation of leukocyte cell‐cell adhesion, which are critical for T cell biological function. Similarly, genes upregulated in the BTP‐2 group were involved in pathways related to the cellular defense response, leukocyte chemotaxis, integrin‐mediated signaling pathway, and leukocyte migration. In particular, cellular metabolism‐related pathways (“response to oxygen levels,” “carboxylic acid biosynthetic process,” “NAD biosynthetic process,” “canonical glycolysis,” and “glucose catabolic process to pyruvate”) were downregulated in BTP‐2‐treated CAR‐T cells (Figure 6D). Gene set enrichment analysis (GSEA) further confirmed that glycolytic processes were significantly downregulated in the BTP‐2‐treated group (Figure 6E). It has been reported that SOCE, calcineurin, and NFAT control cell cycle entry and proliferation in activated T cells through upregulation of glycolysis and oxidative phosphorylation.^[^ ^11^ ^]^ To verify whether the effect of the SOCE inhibitor BTP‐2 on the exhaustion and differentiation of CAR‐T cells could be attributed to the SOCE‐calcineurin‐NFAT pathway, we detected the expression of cytoplasmic and nuclear NFATc2. We found that BTP‐2 and FK‐506 (calcineurin/NFAT inhibitor), but not BAPTA‐AM, both blocked the transport of NFAT from the cytoplasm to the nucleus (Figure 6F,G). We then used FK‐506 to treat CAR‐T cells exposed to persistent tumor antigen for 3 days, and found that the FK‐506 treatment also afforded a strong inhibitory effect on CAR‐T cell exhaustion and terminal differentiation (Figure 6H). In addition, we assessed the Embden–Meyerhof‐pathway (EMP) metabolites and found that the BTP‐2‐treated and FK‐506‐treated CAR‐T cells both exhibited a substantial reduction in the expression of metabolites related to glycolysis, including 3‐P‐glycerate, DHAP, fructose‐1,6‐BP, glucose‐6‐P, L‐lactate, PEP, and pyruvate, suggesting that the effect of the SOCE inhibitor on CAR‐T cells was mediated by the inhibition of glycolysis (Figure 6I). Quantitative PCR results also revealed that the levels of glycolysis‐related genes (*ENO1*, *PGK1*, *SLC2A3*, and *HK2*) in the BTP‐2‐treated and the FK‐506‐treated groups were both lower than those in the control group (Figure 6J). Finally, we compared the glycolytic function of control, BTP‐2‐treated, and FK‐506‐treated CAR‐T cells using Seahorse assays. BTP‐2‐ and FK‐506‐treated CAR‐T cells exhibited significantly decreased glycolysis, glycolytic capacity, and glycolytic reserve (Figure 6K).

## 3 Discussion

Several lines of evidence suggest that exhaustion limits the potency of CAR‐T cells,^[^ ^4a,b,g^ ^]^ whereas less differentiated T cell subsets and memory features are frequently associated with increased antitumor potency and persistence in adoptively transferred T cells.^[^ ^4^, ^18^ ^]^ In this study, we identified cytosolic calcium as an indicator of T cell activation, which can also be used to distinguish CAR‐T cell exhaustion and differentiation. Furthermore, our results extend these findings by demonstrating that T cell exhaustion and the terminal differentiation state, caused by either tonic signaling or stimulation by tumor antigens, can be prevented or reversed by inhibiting the SOCE‐calcineurin‐NFAT pathway in CAR‐T cells independent of the CAR costimulatory domain (CD28 or 4‐1BB). This suggests that BTP‐2 treatment during the manufacture of clinical CAR‐T cell products may enhance product efficacy following adoptive transfer. Our work also demonstrated that CAR‐T cells with weakened calcium signals in vivo exhibited a higher proliferative and less exhausted phenotype, thereby enhancing antitumor responses. These findings suggest that the regulatable CAR platforms described herein may enhance CAR‐T cell efficacy as a result of temporal control of CAR‐T cell signaling. Additional studies are warranted to determine whether this approach could be universally applied to all the CAR constructs.

Previous studies have reported that dynamic changes in intracellular calcium concentrations are crucial for the differentiation and effector functions of T cells because they control the activation of signal transduction molecules and transcription factors.^[^ ^19^ ^]^ Ca^2+^ influx through CRAC channels, termed SOCE, triggers various processes in T cells, including the secretion of cytolytic granules and the activation of Ca^2+^‐dependent enzymes such as calcineurin, Erk1/2, and CaMKII, as well as transcription factors such as NF‐*κ*B and NFAT. Dephosphorylation of NFAT results in its translocation to the nucleus, where it cooperates with other transcription factors to promote T cell development, activation, differentiation, effector functions, and exhaustion.^[^ ^20^ ^]^ The effect of FK‐506, which has been extensively studied in T cells, can be attributed to the inhibition of the calcineurin‐dependent activity of NFAT transcription factors.^[^ ^21^ ^]^ In our study, we confirmed that FK‐506 prevented NFAT translocation from the cytoplasm to the nucleus, downregulated the transcription of NFAT target genes (e.g., *IRF4* and *EGR3*), and endowed CAR‐T cells with a less differentiated and less exhausted phenotype. The transcriptional profiles of the BTP‐2 and FK‐506 treatment groups overlapped significantly, which demonstrated that BTP‐2, as an inhibitor of CRAC,^[^ ^22^ ^]^ did not directly interfere with calcineurin, but inhibited the exhaustion and downstream differentiation of CAR‐T cells mediated at least in part through the NFAT pathway. The advantage of CRAC inhibitors is that they afford higher selectivity and lower toxicity than current US FDA‐approved immunosuppressive agents, such as FK‐506 and cyclosporin A. Consistent with this, the major clinical manifestations of patients with CRAC channelopathies are limited to the immune system, skeletal muscle, and ectodermal‐derived tissues,^[^ ^23^ ^]^ which all agrees with the phenotypes observed in mice with targeted disruption of murine CRAC component or regulator proteins Orai1, Stim1, and/or Stim2.^[^ ^24^ ^]^

Upon activation of T cells, a shift toward anabolic metabolism occurs and is characterized by elevated glycolytic activity, fatty acid synthesis, and increased catabolism of amino acids as an energy source.^[^ ^25^ ^]^ Inhibiting glycolytic metabolism by pharmacological approaches, or simply by providing low levels of glucose, was found to promote the generation of long‐lived memory T cells.^[^ ^26^ ^]^ SOCE and calcineurin have been reported to regulate the expression of multiple glucose transporters and glycolytic enzymes, resulting in impaired glucose uptake, glycolytic metabolism, and cell cycle entry in vitro as well as in vivo through adoptive animal transfer models.^[^ ^11^ ^]^ Similar results were found in murine CD4+ and CD8+ T cells isolated from Nfatc1fl/flCd4cre (Nfatc1‐deficient) mice.^[^ ^27^ ^]^ This is consistent with the results of the present study, which showed that inhibiting SOCE or calcineurin ultimately reduced the exhaustion and improved the persistence of CAR‐T cells. Moreover, the metabolites and transcriptome results of the treatment group also revealed significant reductions in EMP metabolite levels and glycolysis‐related gene expression.

Our study had several limitations, which should be addressed in future studies. First, our investigation focused on in vitro culture and the NSG mouse model. It would be informative to extend our investigations to a humanized mouse model or a mouse‐derived CAR‐T model. However, the key points and conclusions of the present study are not dependent upon the further evaluation of such models, which would be very difficult to establish. Second, the optimal clinical application of calcium modulators remains to be established. We hypothesized that pulsed application of FK‐506 in vivo could improve CAR‐T cell expansion, diminish exhaustion marker expression, and enhance functionality. We note that the observation that calcium modulation augments functionality is arguably paradoxical as CAR‐T cell inactivation would be expected to provide periods of unopposed tumor growth and thereby reduce efficacy.^[^ ^28^ ^]^ Our results also show that during the process of tumor cell killing by CAR‐T cells, the simultaneous use of calcium inhibitors weakens the killing effect; this phenomenon has also been reported in a previous study.^[^ ^29^ ^]^ Ultimately, treatment protocols are designed to modulate calcium signaling in a period of culture time, thereby maintaining a consistent antitumor immune response. A recent study found that a 3 day on/4 day off pulsed schedule in vivo reversibly suppressed CAR signaling and finally achieved improved tumor killing effect, whereas administration every other day had no such response. This study demonstrated that rest could enhance CAR‐T cell efficacy by preventing or reversing exhaustion.^[^ ^9a^ ^]^ This also implies that when designing protocols for simultaneous administration during the killing process in vivo, it is important to consider the timing of the administration. In turn, the choice of the opitimal time point for clinical use of this drug will also be critical. Future studies should therefore involve the development of a rigorous clinical trial design based on the results of in vivo administration in mice.

In summary, our findings highlight the role of the SOCE‐calcineurin‐NFAT pathway in CAR‐T cell exhaustion and differentiation, which is attributed to its effects on metabolic pathways, such as glycolysis. Notably, attenuating calcium signaling renders CAR‐T cells resistant to exhaustion, favors the persistence of highly functional cells, and balances the replicative capacity of long‐lived memory cells and the acquisition of effective antitumor function. The resultant enhancement in antitumor potency thus offer considerable potential for clinical application and improved patient outcomes.

## 4 Experimental Section

### Experimental Design

In this study, the authors aimed to assess the mechanism of calcium‐mediated CAR‐T cell exhaustion and terminal differentiation by CAR tonic signaling or tumor antigen exposure in vitro and in vivo. All in vitro experiments were performed more than thrice using T cells from different healthy donors. In mouse experiments, the sample size was chosen empirically based on the results of previous studies. Reporting of mouse studies followed the Animal Research: Reporting of in vivo experiments guidelines. Mice were randomly allocated to the control group and treatment groups prior to T cell infusion. Data analysis was based on objectively measurable data; the investigators were not blinded to group allocation during data collection and analysis.

### Cell Lines and Cell Culture

The Nalm6 cell line (ATCC CRL‐3273) and 293T cell line (ATCC CRL‐1573) were originally obtained from ATCC. In some cases, cell lines were stably transduced with GFP and firefly luciferase.^[^ ^8a^ ^]^ The Nalm6 and luciferase/GFP/Nalm6 cell lines were cultured in RPMI‐1640 (Corning) supplemented with 10% fetal bovine serum (FBS, Gibco), 100 U mL^−1^ penicillin, and 100 µg mL^−1^ streptomycin (Life Technologies). 293T cells were cultured in DMEM (Corning) with 10% FBS (Gibco). None of the cell lines used in this study was included in the commonly misidentified cell line registry. Prior to in vivo experiments, the cells were regularly tested for Mycoplasma using a MycoAlert Mycoplasma detection kit (Lonza) and determined to be negative.

### CAR Construct Design and Lentivirus Production

The CAR construct comprised a single‐chain variable fragment specific for human CD19 (Clone FMC63), preceded by a CD8a leader peptide and followed by the CD8 hinge, 4‐1BB/CD28 costimulatory domain, and CD3z intracellular regions linked to a P2A‐mCherry sequence. Supernatants containing 4‐1BB‐CAR‐ and CD28‐CAR‐encoding lentiviruses were produced through transient transfection of the 293T cell line. In brief, 70% confluent 293T cells in 10‐cm plates were co‐transfected with plasmids encoding the CAR and the envelope proteins psPAX2 and pMD2.G using polyethylenimine (Polysciences). The 48 and 72‐h viral supernatants were collected, combined, centrifuged to remove cell debris, and concentrated by ultracentrifugation at 25 000 rpm for 2 h. Concentrated lentiviral stocks were frozen at −80 °C for future use.

### T Cell Isolation and CAR‐T Cell Production

Peripheral blood was obtained from healthy volunteers (*n* = 3 or more). All blood samples were handled according to the ethical and safety procedures required. Peripheral blood mononuclear cells were isolated by density gradient centrifugation. T cells were purified and stimulated with anti‐CD3/CD28 beads (Life Technologies) at a 3:1 bead‐to‐cell ratio in T cell medium for 24 h prior to lentiviral transduction. Condensed viral supernatant was used for transduction in accordance with a multiplicity of infection of 2. Transduced T cells were cultured and expanded in RPMI 1640 with 10% FBS, 100 U mL^−1^ penicillin, 100 µg mL^−1^ streptomycin, and 200 IU mL^−1^ IL‐2 (Peptech).3 days following transduction, anti‐CD3/CD28 beads were removed using a magnet. The medium was changed every 2 days, and the cells were plated at 10^6^ cells mL^−1^. Unless otherwise indicated, CAR‐T cells were used for in vitro assays or transferred into mice on days 9–12 following activation.

### Cell Proliferation Assays

Cell proliferation was assessed using the CCK8 assay (ab228554, Abcam) following the manufacturer's instructions. Briefly, cells were seeded in triplicate in 96‐well plates at a density of 2000–10 000 cells per 100 µL. The dye solution was added at the indicated time points, and the plates were incubated at 37  °C for 3–4 h before the absorbance was detected at 450 nm using a microplate reader.

### FACS Analysis of Cytosolic Calcium

CAR‐T cells were incubated with Fluo‐4 AM ester (Invitrogen) for 15–60 min at 20–37 °C. Following adequate incubation, the cells were washed with indicator‐free medium and then further incubated for another 30 min to allow complete de‐esterification of intracellular AM esters. The mean fluorescence intensity of bound Fluo‐4 was determined using flow cytometry (Beckman). To detect dynamic change in cytosolic calcium, cells were suspended in PBS with Ca^2+^ (BasalMedia, China) and stimulated with anti‐CD3/CD28 beads.

### Flow Cytometry

The CAR‐T cell surface phenotype was assessed using the following antibodies: anti‐CD4‐APC‐Cy7 (Clone RPA‐T4), anti‐CD8‐PE‐Cy7 (clone SK1), anti‐CD25‐APC (Clone BC96), anti‐CD69‐PE‐CY7 (Clone FN50), anti‐CD69‐APC (Clone FN50), anti‐PD1‐APC (Clone EH12.2H7), anti‐PD1‐PE (Clone EH12.2H7), anti‐TIM‐3‐PE (Clone F38‐2E2), anti‐LAG‐3‐PE‐CY7 (Clone 7H2C65), anti‐LAG‐3‐PE (Clone 7H2C65), anti‐CD62L‐PE (Clone DREG‐56), anti‐CD45RO‐APC (Clone UCHL1), anti‐IL‐2‐APC (Clone MQ1‐17H12), anti‐TNFα‐PE (Clone Mab11), anti‐Granzyme B‐PE (Clone QA16A02), anti‐IFNγ‐APC (Clone 4S. B3), and anti‐Annexin‐V‐APC (Lot B317159) (all from BioLegend); anti‐TER119‐APC‐eFlour780 (Clone TER119) (from eBioscience); and anti‐PD1‐BV421 (Clone EH12.1) and anti‐TIM‐3‐BV650 (Clone 7D3) (both from BD).

All FACS plots presenting CAR‐T cell phenotype data were conducted using gated mCherry/GFP‐positive CAR‐T cells. Positive/negative populations were determined on the basis of the isotype control (IGg1 k, Clone MOPC‐21; Biolegend). Flow sorting was performed with gated mCherry/GFP‐positive CAR‐T cells using a Beckman Moflo Astrios EQ (Beckman).

### Intracellular Cytokine Staining

For intracellular cytokine staining analysis, CAR‐T cells alone or cocultured with target cells at a 1:1 effector‐to‐target (E:T) ratio in medium containing 1× monensin (# 00‐4505‐51, eBioscience) for 5–6 h. Following incubation, intracellular cytokine staining was performed using the Foxp3/Transcription Factor Staining Buffer Set (#00‐5523‐00, eBioscience) according to the manufacturer's instructions using the following antibodies from BioLegend: anti‐Granzyme B‐PE (Clone QA16A02), anti‐IL2‐PE (Clone MQ1‐17H12), anti‐IFN*γ*‐APC (Clone 4S. B3), and anti‐TNF*α*‐APC (Clone Mab11).

### Coculture Killing Assay

The cytolytic ability of CAR‐T cells was evaluated using luciferase‐based cytotoxicity assays. Approximately 4 × 10^4^ target luciferase‐expressing NALM6 cells were co‐incubated with CAR‐T cells for 4 h at E:T ratios of 1:1 or 2:1 using black‐walled 96‐well plates. Triplicate wells were plated for each condition. Mixed cells were harvested and added to a Bright‐Glo Luciferase Assay system (Promega) for 2 min to allow complete cell lysis, and cell viability was measured using a luminometer. Cytotoxicity efficiency was calculated as follows:

(1) $$\begin{array}{c} \mathrm{lysis}\%\;=\frac{\mathrm{Untransduced}\;T\;\mathrm{cell}\;\mathrm{viability}\;-\;\mathrm{CAR}-T\;\mathrm{cell}\;\mathrm{viability}}{\mathrm{Untransduced}\;T\;\mathrm{cell}\;\mathrm{viability}}\;\times 100\% \end{array}$$

### Cytokine Production by ELISA

Approximately 1 × 10^6^ CAR‐T cells were cultured in 1 mL of medium (without IL‐2 supplementation) in 12‐well flat‐bottom plates for 48 h. Triplicate wells were plated for each condition. The supernatants were collected and analyzed using a human ELISA kit (MLUTI SCIENCES, China). ELISAs were performed using purified anti‐IL‐2, anti‐IFN‐*γ*, anti‐TNF‐*α*, and anti‐Granzyme B mAbs as capture Abs; the corresponding biotinylated anti‐IL‐2, anti‐IFN‐*γ*, anti‐TNF‐*α*, and anti‐Granzyme B mAbs; HRP‐conjugated streptavidin (Sigma Aldrich); and tetramethylbenzidine microwell peroxidase substrate and stop solution (Kirkegaard and Perry Laboratories) according to the manufacturer's instructions.

### Western Blots

Cytoplasmic and nuclear proteins were separated using NE‐PER Nuclear and Cytoplasmic Extraction Reagents kits (#78833, Thermo). Cell lysates were examined by western blot analysis using the Pierce BCA Protein Assay Kit (#23225, Thermo Scientific). The following primary antibodies were purchased from Cell Signaling Technology: anti‐NFAT1 (D43B1), anti‐Lamin B1 (D4Q4Z), and anti‐*β*‐Actin (D6A8) antibodies.

### Real‐Time PCR

mRNA was extracted from the control and treated CAR‐T cell groups using an RNA isolation kit (Qiagen) according to the manufacturer's protocol. Reversed transcription of cDNA was performed using the Super Script First‐Strand Synthesis System (Life Technologies). All reactions were performed with the TaqMan Fast Universal PCR Master Mix (Applied Biosystems) on an Applied Biosystems Step One Plus real‐time PCR machine using the following primers: *PRDM1* (F: AAGCAACTGGATGCGCTATGT, R: GGGATGGGCTTAATGGTGTAGAA), *IRF4* (F: GAGTCACCTGGAATCTTGGC, R: CCTGCAAGCTCTTTGACACA), *BATF* (F: TATTGCCGCCCAGAAGAGC, R: GCTTGATCTCCTTGCGTAGAG), *NR4A1* (F: ATGCCCTGTATCCAAGCCC, R: GTGTAGCCGTCCATGAAGGT), *NR4A2* (F: GTTCAGGCGCAGTATGGGTC, R: CTCCCGAAGAGTGGTAACTGT), *ENTPD1* (F: AGGTGCCTATGGCTGGATTAC, R: CCAAAGCTCCAAAGGTTTCCT), *ENO1* (F: AAAGCTGGTGCCGTTGAGAA, R: GGTTGTGGTAAACCTCTGCTC), *PGK1* (F: TGGACGTTAAAGGGAAGCGG, R: GCTCATAAGGACTACCGACTTGG), *SLC2A3* (F: GCTGGGCATCGTTGTTGGA, R: GCACTTTGTAGGATAGCAGGAAG), and *HK2* (F: AGCCCTTTCTCCATCTCCTT, R: AACCATGACCAAGTGCAGAA).

### mRNA Library Construction and Sequencing

Healthy donor T cells were prepared as described. Total mRNA was isolated and purified from >1 × 10^6^ bulk CAR‐T cells in each group using TRIzol reagent (Invitrogen, Carlsbad, CA, USA) following the manufacturer's instructions. The RNA integrity was assessed using a Bioanalyzer 2100 (Agilent, CA, USA) with RIN number >7.0, and confirmed by electrophoresis with denaturing agarose gel. Poly (A) RNA was purified from 1 µg total RNA using Dynabeads Oligo (dT)25‐61005 (Thermo Fisher) using two rounds of purification. Then the poly(A) RNA was fragmented into small pieces using the Magnesium RNA Fragmentation Module (#e6150, NEB) at 94 °C 5–7 min. The cleaved RNA fragments were reverse‐transcribed to create the cDNA using SuperScript II Reverse Transcriptase (Invitrogen, cat. 1896649, USA), which were next used to synthesize U‐labeled second‐stranded DNAs with *Escherichia coli* DNA polymerase I (#m0209, NEB), RNase H (#m0297, NEB), and dUTP Solution (#R0133, Thermo Fisher). An A‐base was then added to the blunt ends of each strand, preparing them for ligation to the indexed adapters. Each adapter contained a T‐base overhang to ligate the adapter to the A‐tailed fragmented DNA. Single‐ or dual‐index adapters were ligated to the fragments, and size selection was performed with AMPureXP beads. After the heat‐labile UDG enzyme (#m0280, NEB) treatment of the U‐labeled second‐stranded DNAs, the ligated products were amplified by PCR under the following conditions: initial denaturation at 95 °C for 3 min; 8 cycles of denaturation at 98 °C for 15 s, annealing at 60 °C for 15 s, and extension at 72 °C for 30 s; and a final extension at 72 °C for 5 min. The average insert size of the final cDNA library was 300 ± 50 bp. Finally, the 2 × 150 bp paired‐end sequencing (PE150) was performed on an Illumina Novaseq 6000 (LC‐Bio Technology Co., Ltd.) following the vendor's recommended protocol.

### Sequence Alignment and Gene Expression Analysis

Sequenced reads were aligned to the human reference genome (GRCh38) using the HISAT2 software (version 2.0.4).^[^ ^30^ ^]^ StringTie (v 1.3.4d.Linux_x86_64)^[^ ^31^ ^]^ was used to estimate the expression levels of all transcripts. PCA and differential expression analysis were performed using the edgeR package (v 3.26.8) in R (v 3.6.0).^[^ ^32^ ^]^ The DEGs with absolute log_2_(fold change) >2 and P value < 0.05, were considered as significant. GO annotation and GSEA were carried out using the clusterProfiler package (v 3.12.0)^[^ ^33^ ^]^ with default settings. The data of short‐term or long‐term cultured CAR‐T cells are available in the Gene Expression Omnibus (GEO) (accession code GSE178570). The data of CAR‐T cells treated with different drugs are available in the GEO (accession code GSE178998).

### Metabolomic Analysis

CAR‐T cells were produced and cultured as described. Cells in the different groups were collected and counted. Following centrifugation, the cells were kept in liquid nitrogen for 30 s and stored at −80 °C. Metabolites were then immediately extracted and subjected to targeted metabolomic analysis of central carbon metabolism. The samples were vortexed for 1 min after adding 1000 µL precooled MeOH/H2O (3/1, v/v). The samples were precooled in dry ice, freeze‐thawed three times in liquid nitrogen, vortexed for 30 s, sonicated for 15 min in an ice‐water bath, then vibrated at 4 °C for 15 min, followed by incubation at −40 °C for 1 h and centrifugation at 12 000 rpm (RCF = 13 800 × g, *R* = 8.6 cm) at 4 °C for 15 min. An 800 µL aliquot of the clear supernatant was collected and dried by centrifugation. Then, the residue was reconstituted using 150 ultrapure water. Reconstituted samples were vortexed prior to filtration through a centrifuge tube filter, and were subsequently transferred to inserts in injection vials for HPIC‐MS/MS analysis. Electrospray ionization mass spectrometry was conducted by Biotree using a SCIEX 6500 QTRAP+ triple quadrupole mass spectrometer in negative mode. The final concentration (CF, nmol L^−1^) equals the calculated concentration (CC, nmol l^−1^) multiplied by the dilution factor (Dil).

### Seahorse XF Assays

CAR‐T cells were resuspended in Seahorse XF Assay Medium (Agilent Technologies) and seeded at 6000 cells per well in a 96‐well plate. The cell culture microplate (101085‐004, Agilent) was pre‐coated with Cell‐Tak (354240, Corning) for 20 min at room temperature. The XF 96 sensor cartridge was hydrated with 200 µL of calibration buffer per well in a 37 °C non‐CO2 incubator overnight. The extracellular acidification rate (ECAR) was measured in real time in an XFe96 analyzer using a Seahorse XF Glycolytic Stress Assay Kit (103020‐100, Agilent). The rate of glycolysis under basal conditions was measured following the first addition of glucose (10 mm). The cellular maximum glycolytic capacity was defined as the subsequent increase in ECAR after oligomycin (1.5 mm) injection and glycolytic reserve was determined by subtracting the rate of glycolysis before and after addition of 2‐deoxy‐D‐glucose (50 mm).

### Mice and Murine Xenograft Models

Immunocompromised NOD‐SCID‐Il2rg−/−(NSG) mice were purchased from the Shanghai Model Organisms Center, Inc. All mice were bred, housed, and treated ethically in a specific pathogen‐free facility at the Zhejiang University according to institutional guidelines. 6 to 8‐week‐old female mice were randomly assigned to the treatment groups in this study. The mice were inoculated with 1 × 10^6^ luciferase/GFP/Nalm6 cells by tail vein injection, followed by intravenous injection of 1 × 10^6^ mCherry/CAR‐T cells 5 days later. Leukemia progression was measured weekly by bioluminescence imaging using the IVIS imaging system (IVIS Luminu III, Perkin‐Elmer) after luciferin injection. The values were analyzed using Living Image software (Perkin‐Elmer). Mice were humanely euthanized when they demonstrated signs of morbidity and/or hindlimb paralysis. The mice were randomized to the treatment groups to ensure equal pretreatment tumor burdens, and no mice were excluded prior to CAR‐T cell treatment. The researchers were blinded to the treatment during tumor measurement. Peripheral blood was collected at 8 days following CAR‐T cell administration and analyzed by flow cytometry.

### Statistical Analysis

Data are presented as the means ± standard error of the mean (SEM) unless stated otherwise. All in vitro experiments consisted of three or more biological replicates per experimental group and were represented as individual data points. In vivo studies consisted of five mice per group. Statistical tests were performed on data from independent biological replicates (*n* = 3 biological replicates per in vitro experimental group; *n* = 5 mice per in vivo cohort). No data were excluded from the statistical analyses. For comparisons between two groups, a two‐tailed unpaired Student's *t*‐test was applied. For comparisons among three or more groups, one‐way ANOVA with Dunnett post‐hoc test was applied, as indicated. For in vivo experiments, overall survival was depicted by a Kaplan–Meier curve, and the log‐rank test was used to compare survival differences between the groups. A *p*‐value < 0.05 was deemed as the significance threshold for all analyses. *p* values were calculated as mentioned above with *p ≤ 0.05, **p ≤ 0.01, and ***p ≤ 0.001. Analyses were performed using Prism 8 (GraphPad) software and R (v.3.0.2; R Foundation for Statistical Computing).

## Conflict of Interest

The authors declare no conflict of interest.

## Author Contributions

M.S., X.T., and X.G. contributed equally to this work. H.H., P.Q., and Y.H. designed the study, analyzed and interpreted the data, and wrote the manuscript. M.S. and X.T. designed and performed the experiments, analyzed and interpreted the data, and wrote the manuscript. X.G. analyzed the RNA‐Seq data. H.Z. and Y.H. performed the experiments.

## Supporting information

Supporting Information

## Data Availability

The data that support the findings of this study are available from the corresponding author upon reasonable request.
