# Supplementary material for: Inhibition of Calcium Signaling Prevents Exhaustion and Enhances Anti‐Leukemia Efficacy of CAR‐T Cells via SOCE‐Calcineurin‐NFAT and Glycolysis Pathways
Source: Adv Sci (Weinh). 2022 Jan 14;9(9):2103508. doi: 10.1002/advs.202103508 (PMC8948559; doi:10.1002/advs.202103508)
Supplement: Supplementary file 1 — Supporting Information [file ADVS-9-2103508-s001.pdf]

ADVANCED SCIENCE
Open Access

Supporting Information

for *Adv. Sci.*, DOI: 10.1002/advs.202103508

Inhibition of Calcium Signaling Prevents Exhaustion and Enhances Anti-Leukemia Efficacy of CAR-T Cells via SOCE-Calcineurin-NFAT and Glycolysis Pathways

Mi Shao, Xinyi Teng, Xin Guo, Hao Zhang, Yue Huang, Jiazhen Cui, Xiaohui Si, Lijuan Ding, Xiujian Wang, Xia Li, Jimin Shi, Mingming Zhang, Delin Kong, Tianning Gu, Yongxian Hu, Pengxu Qian,* and He Huang**

Supplementary materials

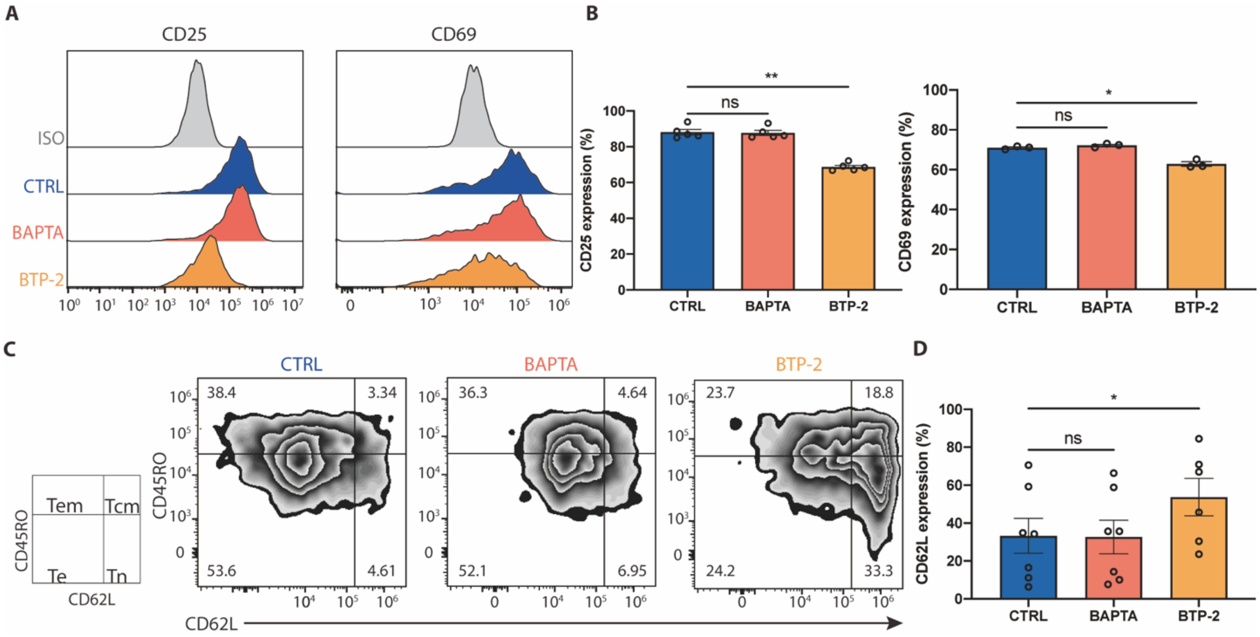

Figure S1. The SOCE inhibitor BTP-2 reduces excessive activation in CD28 CAR-T cells. (**A-D**) Flow cytometric analysis of the expression of (A and B) activation markers and (C and D) memory markers on CAR-T cells. Data are reported as the means ± SEMs. n = 3 or more independent biological replicates, presented as individual points. *P < 0.05, **P < 0.01 and *** P ≤ 0.001 (one-way ANOVA with Dunnett post-hoc test; comparing n = 3 or more *in vitro* biological replicates per group).

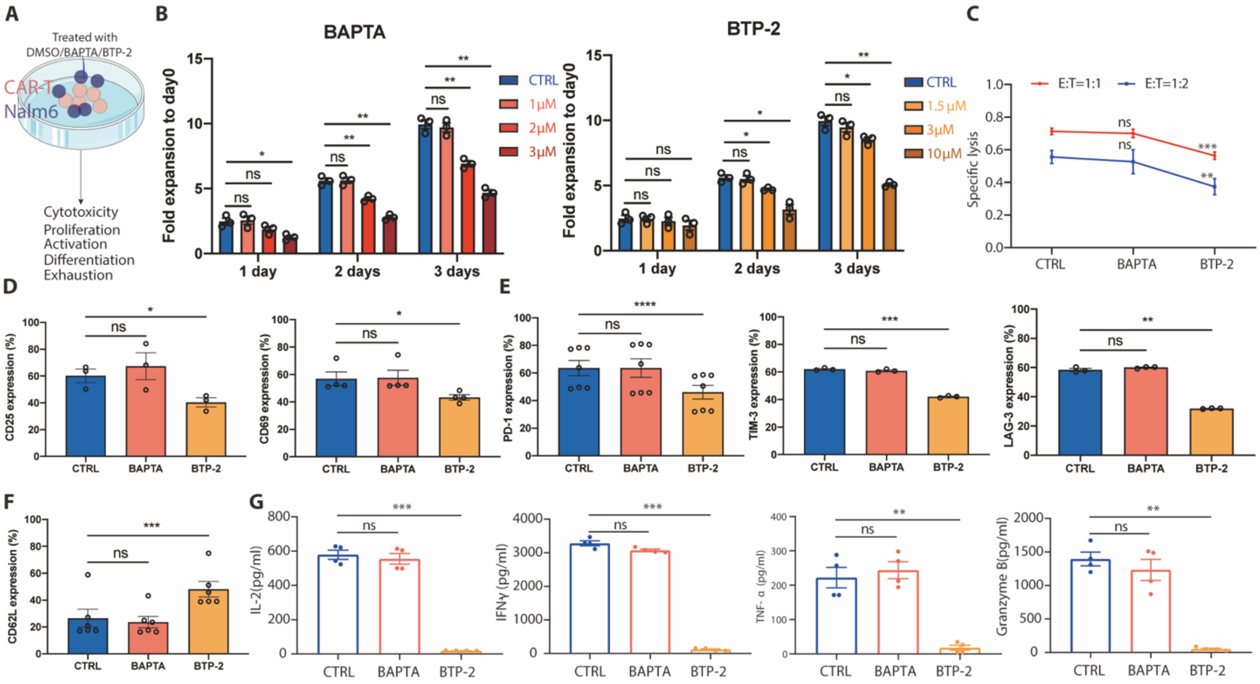

Figure S2. BTP-2 prevents exhaustion of CAR-T cells upon tumor antigen stimulation.

(**A**) Experimental design: CAR-T cells were cocultured with Nalm6 and treated with DMSO/BAPTA /BTP-2 simultaneously for three consecutive days. (**B**) The number of CAR-T cells was calculated using cell counts on days 1, 2, and 3, respectively. (**C**) Lysis of target cells measured at 4 hours. Effector-to-target (E:T) ratios were 1:1 (red line) or 1:2 (blue line). (**D-F**) Flow cytometric analysis of the expression of (D) activation markers, (E)inhibitory receptor markers, and (F) differentiation status (CD62L and CD45RO) markers. (**G)** Secretion of granzyme B, IFN-γ, IL-2, and TNF-α into culture medium was measured using ELISA kits at d3. Data are reported as the means ± SEMs. n = 3 or more independent biological replicates, presented as individual points. *P < 0.05, **P < 0.01 and *** P ≤ 0.001 (one-way ANOVA with Dunnett post-hoc test; comparing n = 3 or more *in vitro* biological replicates per group).

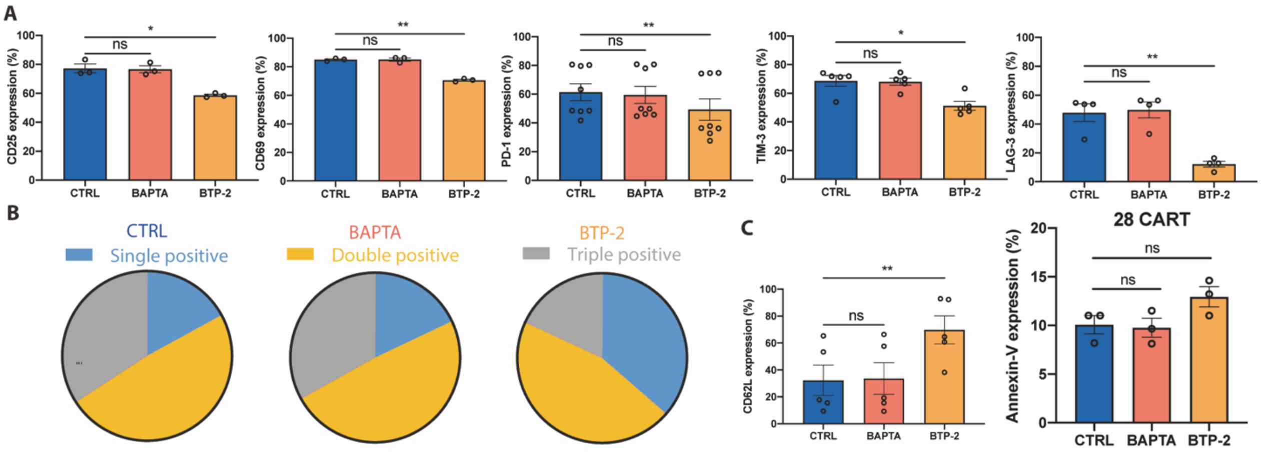

Figure S3. BTP-2 prevents the exhaustion of CD28 CAR-T cells during the prolonged *in vitro* culture.

(A) Flow cytometric analysis of the expression of activation markers (CD25 and CD69) and inhibitory receptor markers (PD-1, TIM-3, and LAG-3). (**B**) Patterns of expression and co-expression of the inhibitory molecules PD-1, TIM-3, and LAG-3. The proportions of non-positive (PD1-TIM3-LAG3-), single-positive (PD1+TIM3-LAG3-, PD1-TIM3+LAG3-, or PD1-TIM3-LAG3+), double-positive (PD1+TIM3+LAG3-, PD1+TIM3-LAG3+, or PD1-TIM3+LAG3+), and triple-positive (PD1+TIM3+LAG3+) cells are depicted. (C) Flow cytometric analysis of the expression of memory markers (CD62L and CD45RO) and Annexin V. Data are reported as the means ± SEMs. n ≥ 3 independent biological replicates, presented as individual points. *P < 0.05, **P < 0.01 and ***P ≤ 0.001 (one-way ANOVA with Dunnett post-hoc test; comparing n ≥ 3 in vitro biological replicates per group).

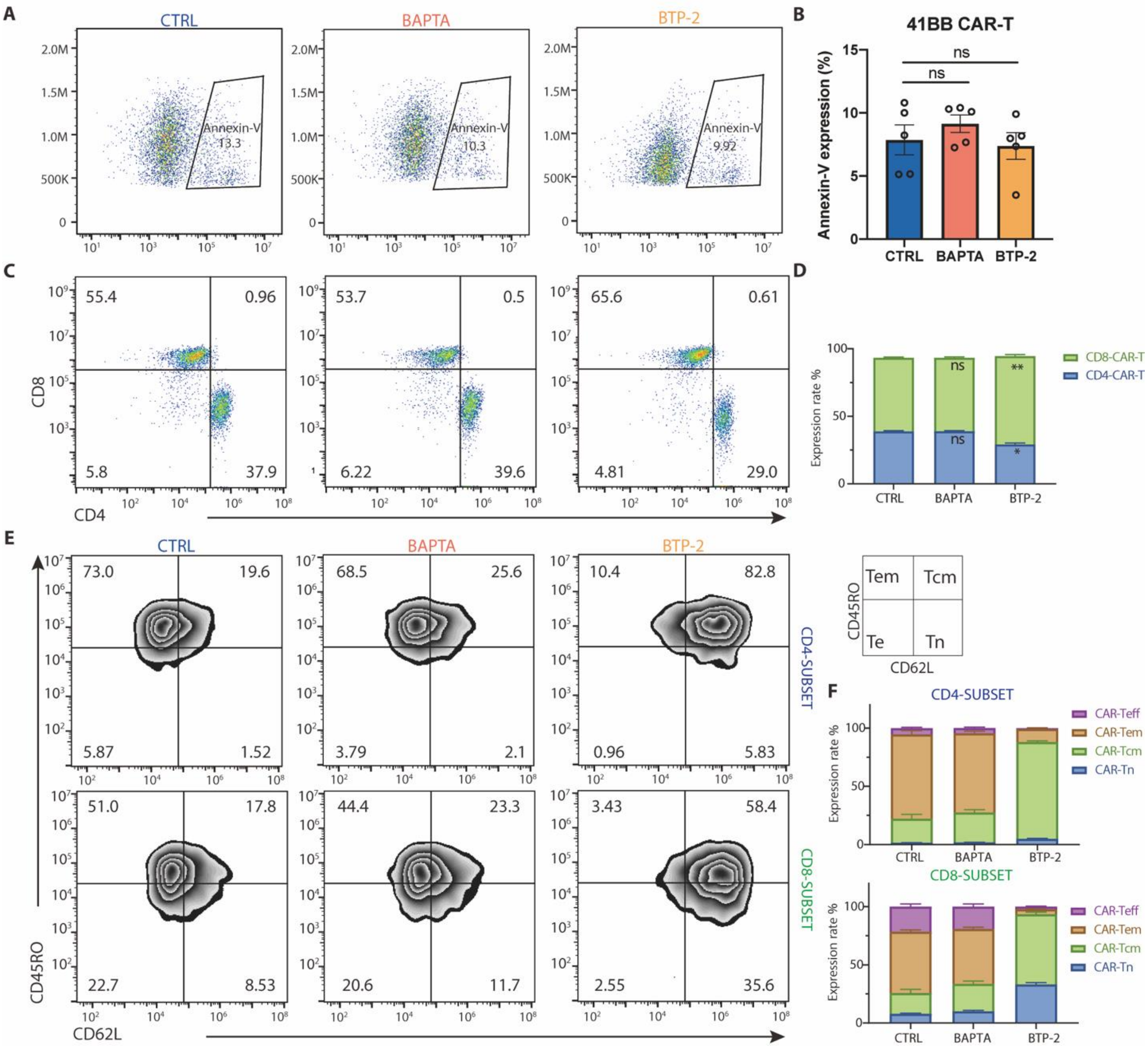

Figure S4. The impact BTP-2 on CAR-T cell apoptosis and CD4/CD8 ratio.

(A-D) Flow cytometric analysis of the expression of (A-B) Annexin V and (C-D) the proportions of CD4 + T and CD8 + CAR-T cells. **（E-F）** Flow cytometric analysis of the expression of memory markers (CD62L and CD45RO) in CD4+ and CD8+ CAR-T cells, respectively. Data are reported as the means ± SEMs. n = 3 or more independent biological replicates, presented as individual points. *P < 0.05, **P < 0.01 and *** P ≤ 0.001 (one-way ANOVA with Dunnett post-hoc test; comparing n = 3 or more *in vitro* biological replicates per group).
